# The Contribution of Endomycorrhiza to the Performance of *Potato Virus Y*-Infected Solanaceous Plants: Disease Alleviation or Exacerbation?

**DOI:** 10.3389/fmicb.2019.00516

**Published:** 2019-03-20

**Authors:** Edyta Deja-Sikora, Louis Mercy, Christel Baum, Katarzyna Hrynkiewicz

**Affiliations:** ^1^ Department of Microbiology, Faculty of Biology and Environmental Protection, Nicolaus Copernicus University, Torun, Poland; ^2^ Centre for Modern Interdisciplinary Technologies, Nicolaus Copernicus University, Torun, Poland; ^3^ INOQ GmbH, Schnega, Germany; ^4^ Faculty of Agricultural and Environmental Sciences, University of Rostock, Rostock, Germany

**Keywords:** *Solanum tuberosum* L., mycorrhiza, *Potato virus Y*, PVY infection, common mycorrhizal network, mycorrhizal transmission of plant viruses

## Abstract

*Solanaceae,* comprising meaningful crops (as potato, tomato, pepper, eggplant, and tobacco), can benefit from a symbiosis with arbuscular mycorrhizal fungi (AMF), which improve plant fitness and support plant defense against pathogens. Currently, those crops are likely the most impacted by *Potato virus Y* (PVY). Unfortunately, the effects of AM symbiosis on the severity of disease induced by PVY in solanaceous crops remain uncertain, partly because the interplay between AMF and PVY is poorly characterized. To shed some light on this issue, available studies on interactions in tripartite association between the host plant, its fungal colonizer, and viral pathogen were analyzed and discussed. Although the best-documented PVY transmission pathway is aphid-dependent, PVY infections are also observed in the absence of insect vector. We hypothesize the existence of an additional pathway for virus transmission involving AMF, in which the common mycorrhizal network (CMN) may act as a potential bridge. Therefore, we reviewed (1) the significance of AM colonization for the course of disease, (2) the potential of AMF networks to act as vectors for PVY, and (3) the consequences for crop breeding and production of AM biofertilizers.

## Introduction: A Global Problem of *Potato Virus Y*

As the global food demand is constantly raising, the development of more efficient and environmental friendly food production approaches is the biggest challenge for modern agriculture. Determining the optimal usage of current agricultural resources seems to be the only way to protect food supply for the human population in near future. However, food security in some parts of the world is severely impacted by losses in arable land due to climatic change and environmental degradation as well as pest damage to economically important crops (Sundström et al., 2014). Among the most devastating pests, the *Potato virus Y* (PVY) belonging to the genus *Potyvirus* within the Potyviridae family represents a serious threat due to high incidence and worldwide distribution. This phytopathogen is particularly destructive to solanaceous crops, i.e. potato (*Solanum tuberosum* L.), tomato (*Solanum lycopersicum* L.), pepper (*Capsicum* spp.), eggplant (*Solanum melongena* L.), and tobacco (*Nicotiana tabacum* L.) (Moury et al., 2017).

PVY exerts the highest economic impact on potato, as it is the third most consumed food crop after rice and wheat (Devaux et al., 2014; ICP, 2018). This was probably the main motivation for conducting many studies on PVY biological and serological variability, using the “potato” virus strains (Blanchard et al., 2008; Lacomme et al., 2017). Once infected with PVY, the potato plant can develop primary morphological symptoms of the disease in timeframe as short as 3–5 days in hypersensitive varieties or later than 2 weeks in more resistant ones (Baebler et al., 2011; Otulak and Garbaczewska, 2014). Generally, symptoms of PVY-induced potato disease (reviewed in details elsewhere; see Glais et al., 2017) are variable, and their severity depends on several factors, i.e. host susceptibility, host growth stage, virus strain/subtype, and environmental conditions (Fox et al., 2017). In some cases, the effect of PVY in the plant is symptomless, which is called latent infection. However, the virus often causes foliage defects that are easily identified visually, e.g. leaf deformation, yellowing, mottling, mild-to-severe mosaic spots, leaf necrosis, leaf drop, but also more or less severe plant stunting (Glais et al., 2017). Necrotic strains of PVY induce the development of potato tuber necrotic ringspot disease, dramatically reducing the quality and quantity of tubers (Glais et al., 2017).

The economic losses caused by PVY are quite considerable. According to recent estimations, PVY is able to affect up to 50% of potato crops in China, which is the world’s largest potato producer (Wang et al., 2011). In other parts of the world, average incidences of PVY are around 44% in USA (Gray et al., 2010), nearly 40% in Poland (Hasiów-Jaroszewska et al., 2014), 37% in Kenya (Were et al., 2013), 34% in Canada (Gray et al., 2010), and 16.5% in Ireland (Hutton et al., 2015). Therefore, special attention is put on controlling PVY infections, which is very challenging due to (1) the occurrence of various recombinant strains, (2) their rapid spreading within the host-plant and in the environment, and (3) the translocation of virus particles to the potato tubers, developing into the next generation of virus-positive plants (Davie et al., 2017; Dupuis, 2017).

In this review, after a short summary on the PVY transmission pathways taking potato as a model host (“Transmission Pathways for *Potato Virus Y*”), we dedicate our attention to ecological function of arbuscular mycorrhizal fungi (“Arbuscular Mycorrhizal Fungi: Ecosystem Service and Biocontrol of Plant Pathogens”) and then concentrate on its role in PVY biocontrol in potato, which is discussed in the light of virus-plant interactions in *Solanaceae* (“The Interactions Between AMF and Viruses in Potato and Other *Solanaceae*”). Finally, we propose the emerging hypothesis on the role of the mycorrhizal soil networks in PVY spreading (“Do AMF Participate in PVY Transmission?”).

## Transmission Pathways for *Potato Virus Y*

**Figure1:**
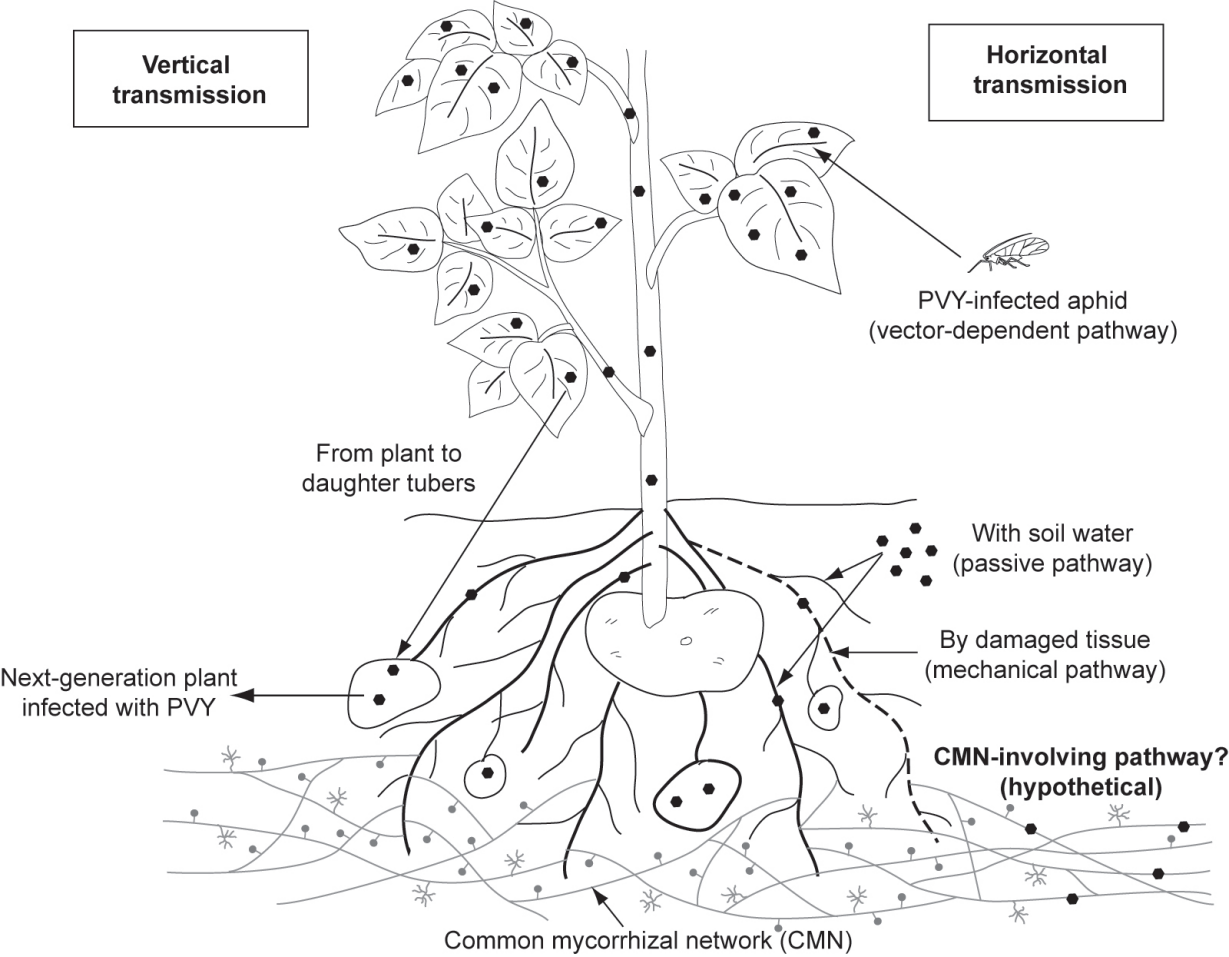
Documented and hypothetical transmission pathways for *Potato virus Y*. PVY was proved to be transmitted by aphid vector, mechanically (by damaged tissue) and passively (with soil water). The pathway involving common mycorrhizal networks, which is proposed in this review, remains hypothetical and requires further consideration.

Plant viruses are transmitted (1) vertically, i.e. from infected plant to the progeny (mother-to-child transmission involves both sexual and asexual propagation *via* seeds, tubers, and cuttings) or (2) horizontally, i.e. mechanically and by many vectors, including insects, soil-borne zoosporic parasitic fungi and protists, nematodes, and mites (Bragard et al., 2013; Blanc and Michalakis, 2016; Lacomme et al., 2017). Among these vectors, aphids, whiteflies, mites, and plasmodiophorids are confirmed to contribute to *Potyviridae* spreading (Bragard et al., 2013). However, aphids are the only identified carrier for members of genus *Potyvirus* (Figure 1).

PVY, like other potyviruses, is transmitted to the host plant primarily *via* insect-dependent pathways by more than 40 species of aphids (Davie et al., 2017). Among them, potato-colonizing aphids (e.g., Myzus persicae, Macrosiphum euphorbiae, and Aphis nasturtii) are proven to be most efficient at disseminating viral pathogens in a non-persistent manner (Nanayakkara et al., 2012). Insects acquire virus within seconds or minutes during probing of PVY-infected epidermal cells. The *Potyvirus*-encoded helper component (i.e., HC-Proteinase, HC-Pro) acting as a “molecular bridge” mediates reversible retention of virions in the insect’s mouthparts. When aphids feed on a healthy plant, PVY particles are released from the stylet to inoculate the tissue (Zhang et al., 2013; Whitfield et al., 2015; Lacomme et al., 2017). Once uncoated, viral genomes replicate in the plant cell, then move through plasmodesmata to the other cells, and finally with phloem sap throughout the whole plant including daughter tubers, which results in a systemic infection. Such PVY-positive seed tubers are important donors of virus to potato crops in the next season (Lacomme et al., 2017).

Potato non-colonizers that casually visit the potato fields, e.g. *Myzus cerasi*, *Aphis glycines*, and *Rhopalosiphum padi*, may additionally serve as PVY vectors. However, they are reported to infect plants less efficiently (Nanayakkara et al., 2012). There is also limited information on the insect-independent way having minor contribution to pathogen spreading. Fageria et al. (2015) indicated that potato may acquire PVY due to mechanical wounding and sap exchange between healthy plants and infected ones. Furthermore, it was suggested that PVY-contaminated water may possibly serve as alternative infectious factor (Mehle and Ravnikar, 2012; Mehle et al., 2014). However, the knowledge on water-dependent transmission of PVY still remains poor.

As potato cultivation is done by vegetative propagation, vertical transfer of PVY is an important source of secondary infection. Planting of PVY-positive tubers may result in massive outbreak of the virus under the field condition. It was reported that the rate of PVY incidence raised almost four times in crops when seed potatoes from PVY-affected field were used for planting (Lacomme et al., 2017). To prevent the transmission of virus through generations and minimize the level of virus incidence, a prophylactic strategy is commonly applied. The quality of potato tubers is strictly monitored, and only certified PVY-free lots are used by growers for potato production in developed countries (notably in Europe and North America) (Gray et al., 2010). Additionally, PVY is also graft-transmissible [i.e., by joining pieces of infected plants with healthy ones; (Lacomme et al., 2017)]; however, this way of virus spreading does not play a significant role in potato production.

We hypothesize the existence of an additional transmission pathway for PVY involving mycorrhizal networks created by hyphae of arbuscular mycorrhizal fungi (Figure 1). The question whether hyphal networks may contribute to the spreading of PVY is detailed in “Do AMF Participate in PVY Transmission?,” after the ecological role of arbuscular mycorrhizal fungi is discussed.

## Arbuscular Mycorrhizal Fungi: Ecosystem Service and Biocontrol of Plant Pathogens

Arbuscular mycorrhizal fungi (AMF), including species such as *Rhizophagus irregularis* syn. *Glomus intraradices* and *Funneliformis mosseae* syn. *Glomus mosseae,* are members of the phylum Mucoromycota and subphylum Glomeromycotina. They are obligate biotrophs ubiquitously distributed in soils. AMF form symbiotic associations, called arbuscular mycorrhiza, with roots of nearly 74% of all plant species, including agricultural ones (van der Heijden et al., 2015). Highly dense extraradical hyphal networks of AMF extend into the soil outside of the rhizosphere and are able to uptake soil mineral compounds, such as phosphate, nitrogen, potassium ions, and sulfate, that are physically and chemically less available for plants (Wang et al., 2017). These nutrients are transferred *via* the mycelium to arbuscules, which are highly branched tree-shape structures within root cortical cells functioning as exchange interface between partners (Walder et al., 2016). Host plants provide AMF with a habitat: a physical support and a favorable physiological environment that ensures easily accessible energy source in the form of sugars and lipids (Rich et al., 2017). In agriculture, the endomycorrhizal association is known to increase plant health and fitness, thus being a crucial ecosystem service provider (Gianinazzi et al., 2010). Beneficial aspects of arbuscular mycorrhiza include improvement of the plant nutritional status, biomass, and tolerance to abiotic environmental stresses, e.g. salinity or drought (Jacott et al., 2017; Basu et al., 2018). Furthermore, the AMF-plant root symbiosis has the capacity to protect the plant from pathogen attack and enhance the ability of pathogen-infected plant to resist the disease (Jacott et al., 2017). In particular, there is plenty of evidence indicating that arbuscular fungi exhibit great potential for biocontrol of many different phytopathogens, including viral ones (Whipps, 2004; Singh and Giri, 2017), by modulating the multitrophic interactions and stimulating plant defense responses.

Several protective mechanisms against phytopathogens are involved in mycorrhizal systems, which include the promotion of plant growth, reduction of colonization sites available for attackers, alteration in root morphology, increase in damage compensation, changes in both root exudate and rhizosphere microbiome composition, and eventually activation of plant immune system (Whipps, 2004; Singh and Giri, 2017). A number of reports provide evidence on the phenomenon of mycorrhiza-induced plant resistance (MIR), that is a complex response depending on hormonal crosstalk and resulting in systemic protection against various pathogens (Pozo and Azcon-Aguilar, 2007; Jung et al., 2012). It is proposed that the MIR involves three processes: (1) systemic priming of salicylic acid-dependent genes, (2) increased production of abscisic acid, in order to promote cell wall defense, and (3) priming of jasmonate- and ethylene-dependent defense pathways (Cameron et al., 2013). Symbiosis-mediated systemic protection is observed in the below- and aboveground parts of the plant. This defense mechanism was proved to confer against infections with many different biotic factors (e.g., soil-borne fungi and nematodes) and to reduce the incidence of enemy attack (e.g., insect) (Jung et al., 2012).

Currently, large body of articles is focused on bioprotective function of mycorrhiza and AMF-mediated phytopathogen biocontrol (Singh and Giri, 2017), while relatively little is known about the potential contribution of AMF to increased plant susceptibility to pathogen infection. Several reports indicate much higher sensitivity of mycorrhizal plants to different shoot attackers, e.g. parasitic fungi, aphids, and viruses (Gange et al., 1999; Gernns et al., 2001; Xavier and Boyetchko, 2004; Hartley and Gange, 2009). Although these studies demonstrate the positive correlation between mycorrhiza and more rapid manifestation of plant disease, the role of AMF in the “stimulation” of phytopathogens is most likely indirect, and results from the modified physiology of shared host (Borowicz, 2001). Gernns et al. (2001) showed that *Glomus etunicatum*-inoculated barley plants were more susceptible to the fungal pathogen *Erysiphe graminis*. Kamińska et al. (2010) indicated that *G. mosseae* increased the severity of disease caused by phytoplasma strains (class *Mollicutes*) to periwinkle plants. Furthermore, Nemec and Myhre (1984) found *G. etunicatum*-associated sour orange and Duncan grapefruit seedlings to be strongly affected by *Citrus tristeza virus* and *Citrus leaf rugose virus*, respectively.

Surely, as an active participants of symbiosis, mycorrhizal fungi display the ability to influence the interaction between plant and its enemies. Abovepresented data show that AMF can play a dual role in plant pathogenesis; however, the factors (e.g., environmental conditions) or processes (e.g., additional molecular events related to plant defense priming) underlying these observations are not clear. In such multi-species relationship, which involves both mutualistic (plant-AMF) and antagonistic (plant-enemy) interactions, the specific outcome of AMF-phytopathogen interaction is difficult to predict, thus each experimental system should be treated individually.

## The Interactions Between AMF and Viruses in Potato and Other *Solanaceae*

There is a gap in the knowledge of the direct AMF-PVY interaction in potato plant. For this reason, in this section, we discuss data for experimental settings with different solanaceous host plants, various species of arbuscular fungi, and different viruses (summarized in Table 1). The studies mentioned below describe the positive effects of AMF on virus-infected plants before the negative interactions in tripartite systems are presented.

**Table 1:** Interactions between solanaceous plants, symbiotic AMF, and pathogenic viruses.

Host plant	Virus and strain (if known)	AM fungus	Mycorrhiza before viral infection	Observation	References
**Plant disease alleviation**					
Potato cv. Pirol	*Potato virus Y* (PVY)	*G. intraradices*	No	Improved plant growth	Thiem et al., 2014
Tomato	*Tomato yellow leaf curl Sardinia virus* (TYLCSV)	*F. mossae*	Yes	Lower titer of virus in plant tissue	Maffei et al., 2014
**Plant disease exacerbation**					
Potato cv. Marfona	*Potato virus Y* (PVY)	*G. intraradices*	No	Increased activity and concentration of virus Reduced potato growth	Sipahioglu et al., 2009
Tomato	*Tomato aucuba mosaic virus* (TAMV)	*G. macrocarpum*	Yes	Increased concentration of virus	Daft and Okusanya, 1973
Tomato	*Potato virus X* (PVX)	*G. macrocarpum*	Yes	Increased concentration of virus	Daft and Okusanya, 1973
Tomato	*Tobacco mosaic virus* (TMV)	*Glomus* sp.	Yes	Increased concentration of virus	Jabaji-Hare and Stobbs, 1984
Tomato	*Tomato spotted wilt virus* (TSWV)	*F. mossae*	Yes	Upregulation of fewer defense genes Increased plant sensitivity	Miozzi et al., 2011
Tobacco	*Tobacco mosaic virus* (TMV)	*G. intraradices*	Yes	Earlier and more severe foliar disease	Shaul et al., 1999

Significant attenuation of virus-induced plant disease following mycorrhizal establishment was shown in two research papers (Maffei et al., 2014; Thiem et al., 2014). Maffei et al. (2014) demonstrated the alleviation of the symptoms and lower titer of *Tomato yellow leaf curl Sardinia virus* (TYLCSV) infection in tomato previously colonized by *F. mosseae* compared to non-inoculated plants. Moreover, virus exerted no effect on the level of root colonization by AMF maintained after the TYLCSV infection. Similarly, Thiem et al. (2014) noticed improved plant growth of PVY-infected potato inoculated with *G. intraradices,* although the fungus itself exerted no influence on the growth of virus-free plant. However, the protective role of AMF may occur at some conditions. Slezack et al. (2000) emphasized that stable symbiotic association prior to a pathogen attack is required for displaying protective function of arbuscular mycorrhiza. On the other hand, the study by Thiem et al. (2014) demonstrated that mycorrhiza exerted also its beneficial effect in situation when virus-positive plants were inoculated with AMF.

In contrast, several studies indicated that the symbiosis with AMF promoted multiplication of viral particles in the host plants (Daft and Okusanya, 1973; Jabaji-Hare and Stobbs, 1984; Shaul et al., 1999; Sipahioglu et al., 2009; Miozzi et al., 2011). Daft and Okusanya (1973) noted the increased extraction levels of both *Tomato aucuba mosaic virus* (TAMV) and *Potato virus X* (PVX) from tomatoes (S. lycopersicum) forming mycorrhiza with *Gigaspora macrocarpum* (previously *Endogone macrocarpa*). Jabaji-Hare and Stobbs (1984) used electron microscopic analysis to demonstrate the higher concentration of *Tobacco mosaic virus* (TMV) in roots of tomatoes associated with *Glomus* sp. compared with non-mycorrhizal controls. The similar observation was done by Shaul et al. (1999), who reported earlier onset of symptoms and more severe development of foliar disease (i.e., larger necrotic lesions) in *G. intraradices*-colonized tobacco infected with TMV. The process underlying this phenomenon is not clear yet. However, the authors hypothesized the existence of a AMF-triggered regulatory pathway suppressing plant defense mechanisms, which enhanced sensitivity of AMF-tobacco to viral attack. Furthermore, Sipahioglu et al. (2009) observed that the inoculation of PVY-infected potato with *G. intraradices* increased the virus activity and reproduction rate. This in turn exacerbated the plant disease symptoms, resulting in reduced potato growth. The authors suggested that raised accumulation of PVY following potato mycorrhization might be at least partially attributed to an improved nutrition of the plant. More recently, Miozzi et al. (2011) indicated that symbiotic interaction between tomato and *G. mossae* negatively affected a plant defense response to *Tomato spotted wilt virus* based on transcriptomic analysis. The researchers found differences in gene expression patterns between roots and aboveground parts of mycorrhizal plants. The overall number of genes expressed in the shoot of AMF-colonized tomato decreased upon viral infection in comparison with non-mycorrhizal plants. Additionally, the upregulation of fewer defense genes with simultaneous weaker downregulation of primary metabolism genes was revealed in shoot transcriptome of mycorrhizal tomato. This observation suggested the contribution of AMF to increased plant sensitivity to viral pathogen.

Regarding available studies, it is obvious that AMF are not simply a “meaningless” participant of these tripartite associations, but fungal partner directly and differently contributes to the plant performance upon viral infection. Although most frequently adverse effects of AMF are postulated, it should be highlighted that there are inconsistencies in the results of available studies. These inconsistencies may be partially related to a specific design of abovementioned experiments, in which plant mycorrhization was done only before or only after virus acquisition. Furthermore, the additional factors that may explain such observations include different level of functional compatibility between host plants and AMF species and variable level of mycorrhization under laboratory conditions. Although AMF and host plants can be compatible to form mycorrhiza, the level of their functional compatibility (expressed as the rate of nutrients exchange) may be variable (Ravnskov and Jakobsen, 1995). The functional compatibility between the partners is essential for both, symbiosis effectiveness and beneficial services of mycorrhiza. It influences plant fitness and productivity, which is related to the amount of nutrients transferred from fungus to the host (Ravnskov and Jakobsen, 1995; Walder et al., 2012). Kapulnik et al. (2010) concluded that the selection process for the most suitable AMF inoculant should be oriented toward target host plant or even variety. Singh et al. (2012) demonstrated that the host-AMF compatibility differed between cultivars of durum wheat, as the same fungal strain (*G. intraradices* DAOM 197198) preferentially colonized some specific plant genotypes, while the others were less favored and developed significantly lower level of the symbiosis. Interestingly, the host-AMF compatibility was shown to be partially modified by growing conditions (i.e., soil fertility), which means that this aspect of mycorrhiza is environment-dependent and thus variable even for the same fungus-plant combination. Nevertheless, it is suggested that the bioprotective function of mycorrhiza (e.g., plant disease control) depends on the compatible interaction between the partners (Xavier and Boyetchko, 2004). This may further account for the contradictory effects of mycorrhiza reported in the studies presented above (Table 1).

Thus far, the interaction between potato, PVY and AMF was studied in one setting where mycorrhizal development was established after viral infection (Sipahioglu et al., 2009; Thiem et al., 2014). Therefore, it still remains to be elucidated how AMF affect immune system of healthy plant and whether symbiosis-induced changes in plant defense mechanisms occurring before infection can reduce virus multiplication rate and alleviate disease symptoms.

**Figure 2:**
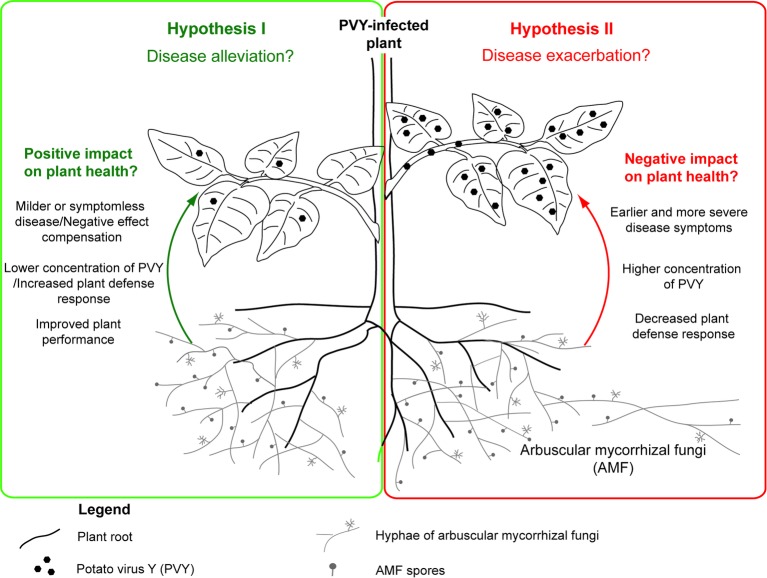
Dual role of common mycorrhizal network in the development of PVY-induced potato plant disease. The positive effects of AMF resulting in disease alleviation (Hypothesis I) are summarized in green panel at the left side, while the negative effects (Hypothesis II) are presented in the red box at the right side.

Interactions in tripartite association between host plant, its symbiotic colonizer – arbuscular mycorrhizal fungus, and viral pathogen remain an interesting research topic, due to limited information on the impact of arbuscular mycorrhiza on viral infection and disease development. Conclusions based on available studies seem to be contradictory, because some researchers indicated alleviating effects and thereby bioprotective function of AMF, whereas others demonstrated the AMF-dependent stimulation of viral activity (Table 1). Due to abovementioned benefits triggered by mycorrhization, we propose a scheme (using potato plant as a model host) in which arbuscular mycorrhizal fungi can protect PVY-infected potato plant by alleviating disease symptoms (Figure 2; Hypothesis I). Nevertheless, this scheme also assumes that, under specific conditions, AMF may exacerbate PVY-induced disease by lowering plant defense response to the virus (Figure 2; Hypothesis II). More studies are needed to understand the physiological basis of PVY severity in mycorrhizal systems, in order to implement optimized strategies when using mycorrhizal products.

## Do AMF Participate in PVY Transmission?

In case of PVY transmission, only insects are well documented to carry this pathogen. However, other hypothetical vectors should not be excluded until additional studies are made. One possible pathway for PVY spreading may involve endomycorrhizal fungi (Figure 1). AMF interconnect many individual plants, of the same or different species, by extraradical hyphal networks (called common mycorrhizal network, CMN) (van der Heijden and Horton, 2009). Compatible mycorrhizal networks may fuse due to anastomoses, thus creating highly dense fungal linkages functioning as underground communication systems in a source-sink relationship manner. A number of studies have shown that mycorrhizal networks provide a route for resource fluxes among plants. CMN allocate nutrients, water, allelopathic substances, and signaling and defense molecules (Barto et al., 2011; Bücking et al., 2016), thus contributing to the plant performance (physiology, survival, adaptation, fitness, competitiveness, and function). However, the role of the CMN in the transfer and eventual release of microorganisms (mainly bacteria) or viruses remains more largely ignored. For this reason, a question is raised whether AMF and CMN may additionally serve as “bridge” for PVY transfer between adjacent potato plants. According to previous indication, virus exchange between host plant and fungal vector depends on the existence of ectoplast-limited thallus (Campbell, 1979). Symbiosis of AMF and host plant relies on an exchange of nutrients across the absorptive structure (arbuscule). Therefore, it can be suspected that plant membrane-bound thalli of endomycorrhizal fungi seem to provide target sites where potential adsorption of virus and its endocytic uptake by the fungus may take place; however, this pathway of virus transfer was not confirmed in the previous study by Jabaji-Hare and Stobbs (1984). Although AMF are not yet demonstrated to be carriers for plant viruses, the other soil-borne obligatory parasitic fungi belonging to Chytridiales and Plasmodiophorales are capable of transmitting plant viruses (Rochon et al., 2004). Some fungal species, e.g. *Synchytrium endobioticum* and *Spongospora subterranea*, were suggested to be implicated in transmission of *Potato virus X* (PVX) and *Potato-mop top virus* (PMTV), respectively (Nienhaus and Stille, 1965; Andersen et al., 2002; EFSA Panel on Plant Health et al., 2018). Nevertheless, in case of PVX, there is no further evidence for fungus-dependent spreading. On the other hand, it is known that AMF can host both endobacteria and mycoviruses within hyphae and spores (Bonfante and Desiro, 2017; Turina et al., 2018). The first observation of viral-like particle was seen in spores of *Scutellospora castanea* (Hijri et al., 2002). Later on, mycoviruses affecting fungal fitness were shown to be transmitted both vertically from hyphae to spores and horizontally *via* hyphal anastomosis between compatible AMF isolates (Kitahara et al., 2014); however, transfer of these virus particles to plants was not confirmed. Nevertheless, our previous study based on macroscopic analysis revealed viral-like structures in the arbuscules of *R. irregularis* associated with PVY-infected potato cells (Thiem et al., 2014). Although interesting, this result should be treated with caution since no additional technique (e.g., immunolocalization assay) was used to confirm the presence of PVY in the mycelium of arbuscular fungus. From this discussion, the hypothesis suggesting that AMF may serve as a bridge for plant viruses should be investigated. Such research topic would enlighten further questions regarding (1) the potential source of infection (e.g., from plant or soil), (2) the process of virus acquisition in AM structures (directly or *via* mechanical wounding), and (3) release (the transfer *via* arbuscules, or late infection from collapsed or decomposing fungal tissue within root or in the rhizosphere). Alternatively, it would be interesting to know if any possible mechanisms, which would prevent plant virus from entering AMF cells, exist. These could be based on a lack of receptors on the AMF wall surface, on a physical protection (fungal wall), or cell death programming to isolate all or part of the infected mycorrhizal structures. This may be achieved (1) by testing detection methods of PVY with molecular and microscopic approaches in mycorrhizal propagules (i.e., spores, extraradical hyphae), (2) by incubating mycorrhizal mycelium with a PVY solution and to try to detect the eventual presence of viral particles (transmission microscopy and molecular tracking) within structures after washing as well as phenotypic observation of the hyphae, and (3) to follow eventual transmission of the virus from infected to non-infected plants interconnected by mycorrhizal mycelium. Ultimately, chemical or biochemical protection from mycorrhizal fungi against viral particles may be conceivable and may be studied by the monitoring of the PVY integrity and infection potential after incubation of viral particles with mycelium exudates or lysates.

## Conclusions

Considering the fragmentary knowledge of AMF-plant-virus interactions, the questions regarding potential contribution of AMF to PVY-induced disease development or even transmission in the frame of the CMN cannot be answered yet. Surely, AMF can play a dual role in PVY infection, which is reflected in the proposed hypotheses (Figure 2); however, specific factors shaping this interaction are not known. Additional studies based on advanced molecular methods (e.g., high-throughput transcriptome sequencing, TEM imaging, PVY-ultrastructure-immunolabeling) are needed to test under what conditions AMF can act as an ally in the mitigation of viral diseases. This may allow the identification of additional environmental factors that modulate the interplay between fungus and virus, therefore resulting in different response of host plant. Acquired knowledge can improve our understanding of ecological relationships and provide new ideas in biocontrol of crop plant pathogens in the future. A vector role of AMF in PVY transmission may be considered, as contaminated mycorrhizal products can be potentially responsible for virus introduction and spreading after plant inoculation. Therefore, there is an urgent need for clarification of the impact of AMF on PVY infection and the expression of symptoms.

## Author Contributions

ED-S prepared the first version of manuscript and graphics, LM, and CB participated in the preparation of the manuscript. KH determined the first concept of the review and participated in the preparation of the manuscript. All authors revised the manuscript and approved the publication.

### Conflict of Interest Statement

The authors declare that the research was conducted in the absence of any commercial or financial relationships that could be construed as a potential conflict of interest.
